# A narrative review of endovascular treatment in addressing arterial and venous erectile dysfunction

**DOI:** 10.3389/fradi.2025.1701606

**Published:** 2025-11-20

**Authors:** Kiara Rezaei-Kalantari, Seyed Mohammad Zamani-Aliabadi, Maryam Jafari, Salah D. Qanadli

**Affiliations:** 1 Department of Radiology, Rajaie Cardiovascular Medical and Research Center, Iran University of Medical Sciences, Tehran, Iran; 2 Department of Medical Physics, School of Medicine, Iran University of Medical Sciences, Tehran, Iran; 3 Department of Radiology, Firouzgar Hospital, Iran University of Medical Sciences, Tehran, Iran; 4 Cardiothoracic and Vascular Division, Department of Diagnostic and Interventional Radiology, Lausanne University Hospital and University of Lausanne, Lausanne, Switzerland

**Keywords:** erectile dysfunction, endovascular, arteria, venous, review

## Abstract

Erectile dysfunction (ED) is a worldwide health concern and clinical condition for men, leading to high medical costs and imposing significant emotional and psychological burdens on sufferers annually. ED is associated with multiple causes, including psychological factors and organic issues such as arterial insufficiency and venous leakage. Endovascular treatments have emerged as promising options for managing ED, offering minimally invasive procedures that can improve blood flow to the penis and restore erectile function. Different endovascular interventional approaches have been implemented with varying success rates and therapeutic impacts, and efforts continue to optimize these methods (both arterial and venous) for maximum effectiveness and minimal invasiveness. This narrative review aims to provide an overview of endovascular treatments for arterial and venous types of ED, discussing their mechanisms of action, efficacy, safety, and future directions.

## 1 Introduction

**Figure 1:**
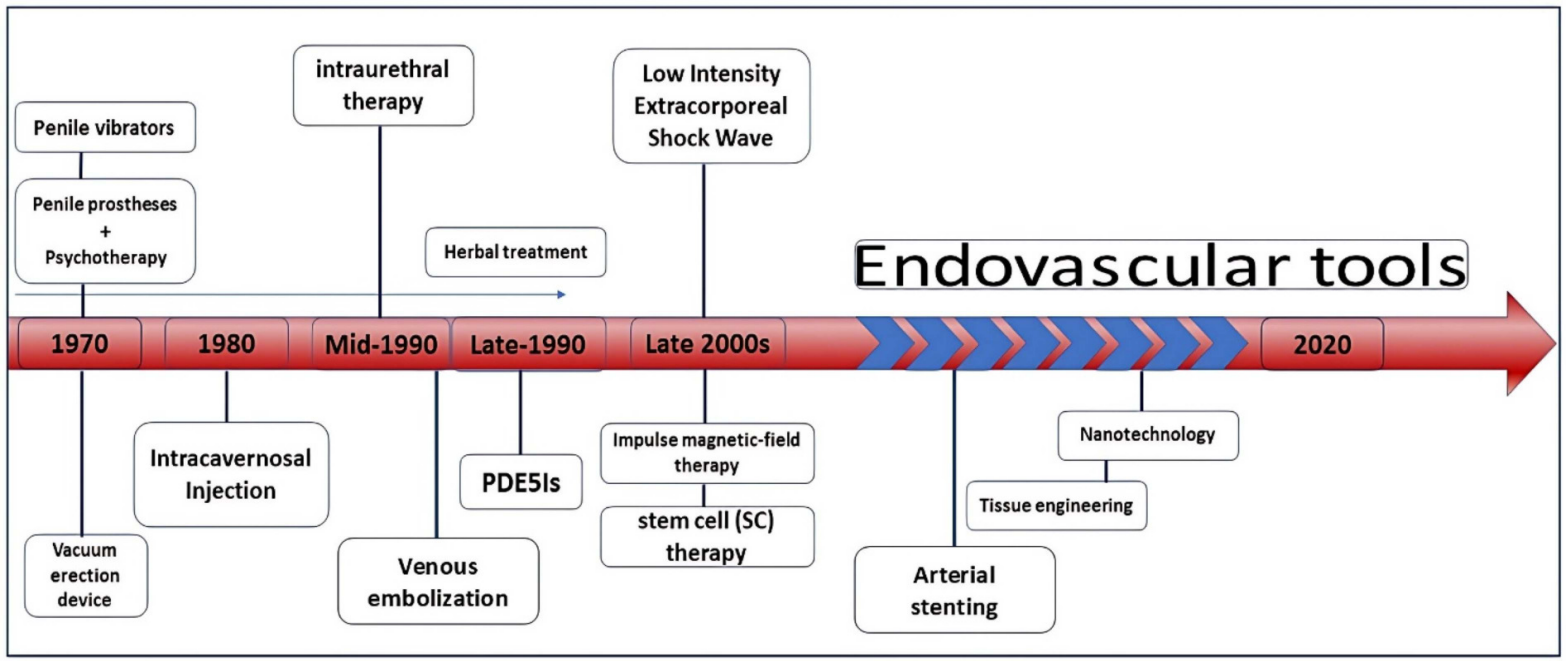
An overview of the applied approaches trend in the ED treatment in recent decades.

Erectile dysfunction (ED) is a significant health issue and a growing global concern. It is characterized by the inability to achieve or maintain an erection sufficient for satisfactory sexual intercourse. Additionally, ED imposes both financial and psychological burdens on societies annually (1, 2). Impotence is a less common equivalent term often confused with other non-medical meanings (3). Since serious studies on this condition began in 1995, its prevalence has increased. According to the latest statistics, the global prevalence among men is estimated to range from 3% to 80%, depending on the methodologies used in different studies (4–6). Nowadays, it is estimated that around 325 million people worldwide are affected by this condition (4). Although ED is widespread, precise figures are not available for all geographic regions (5). Recently, a significant rate of ED was reported in the UK, where approximately a quarter of the 1,000 men surveyed admitted to experiencing ED in half of their sexual encounters (7). Correspondingly, in the United States, the prevalence of ED among men aged 20 years and older is estimated at 18.4%, affecting around 18 million men. Additionally, evidence indicates a 5% increase in ED among sexually active men aged 20–39 years (8). ED can be classified into two main types based on its underlying causes: organic (physical) and psychogenic ED. Organic ED is caused by factors such as vascular, neurological, hormonal, or anatomical abnormalities. A specific subtype of organic ED, vasogenic ED, can be further categorized based on the vascular issues involved, such as arteriogenic ED (due to insufficient arterial blood flow) and venogenic ED (caused by improper venous outflow). On the other hand, psychogenic ED results from psychological factors, such as stress, anxiety, or depression. It is important to note that ED often involves a combination of both psychogenic and organic factors, making it practically impossible to distinguish between these two forms based solely on clinical outcomes (9, 10). Age is strongly associated with ED, with nearly 5% of cases occurring in men in their 40 s and more than 50% of cases in those in their 70 s (11, 12). Treatment is crucial not only because the condition is often considered taboo but also because ED is more common than many people realize and is associated with significant emotional distress and shame (13, 14). Several therapeutic approaches have been introduced to overcome or reduce ED, with some showing promise (15–17) (Figure 1). Currently, the main treatments for ED fall into several categories: oral medications, external supportive devices, and invasive and non-invasive interventions (18). Traditional herbal medicine, particularly indigenous herbs from East Asia, has been used to treat ED due to its natural properties. Studies suggest that these herbs may be more effective than Western medicine, as confirmed by *in vivo* and *in vitro* histopathological research (19–21). However, this treatment method is not universally applicable, and excessive use of plant extracts can have side effects (22, 23). In recent decades, drug treatments for ED have advanced, with new medications being developed and ongoing research aimed at optimizing pharmacokinetics and improving drug delivery. However, underlying conditions such as high blood pressure and diabetes, as well as potential drug interactions, can limit the use of oral medications. Additionally, it is important to consider that some individuals may have contraindications to PDE5 inhibitors and that these medications can be expensive. Innovative approaches, such as endovascular interventions, are now a major focus of research. This narrative review examines the literature on endovascular treatments, including their mechanisms of action, effectiveness, safety, and potential future developments in ED treatment.

### 1.1 Erection physiology and penile anatomy

Anatomically, the penis is attached to the body and consists of a root and a pendulous body. The root includes three bodies of erectile tissue involved in erection: the two crura and the bulb, which are connected to the pubic arch, and the perineal membrane (24). During a penile erection, all three erectile bodies—highly specialized vascular structures—become engorged with blood (25). Blood supply to the penis is provided by the hypogastric iliac artery, which branches from the common iliac artery. This artery supplies blood through the internal pudendal artery and its branches, including the common penile artery. The common penile artery further divides into the bulbourethral artery (supplying the glans and distal urethra), the dorsal artery, and the cavernosal arteries (26).

**Figure 2:**
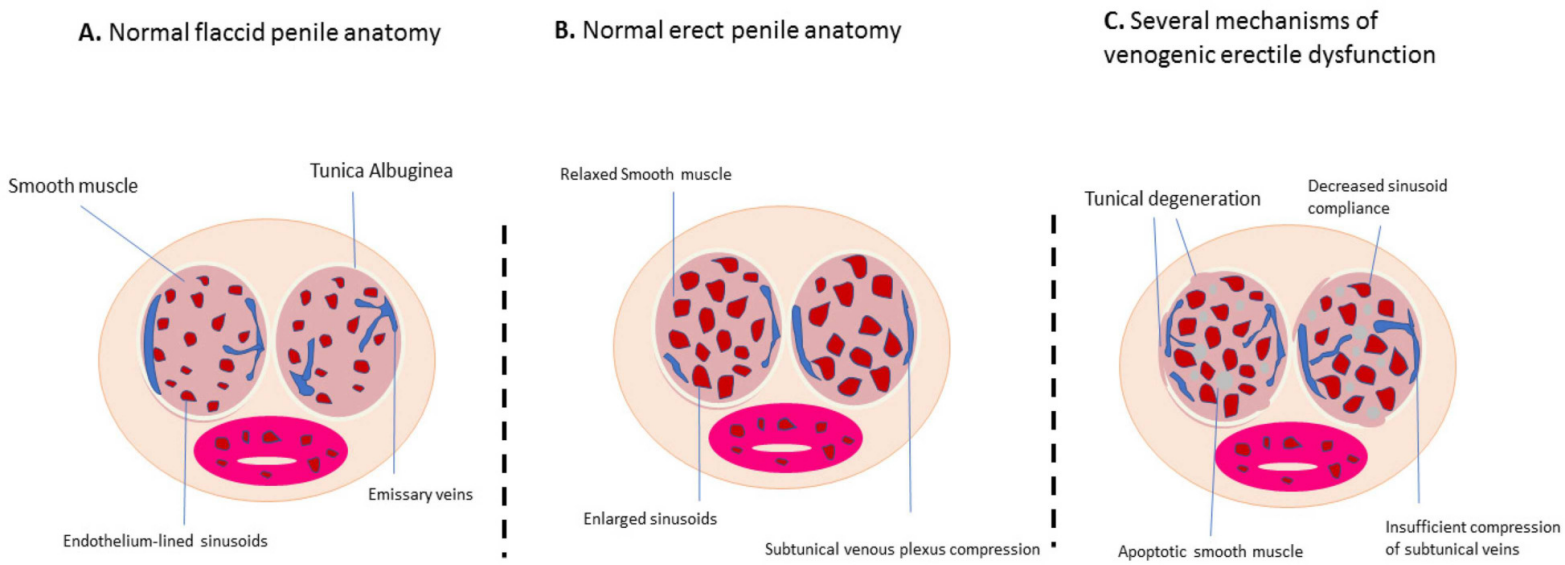
Schematic of the anatomical structure in the normal flaccid state **(A)**, normal erection **(B)**, and erectile dysfunction **(C)** (cross-sectional view of the penis).

Venous drainage of the penis is managed by three groups of veins: superficial, intermediate, and deep (27). The superficial veins primarily drain the penile skin and can connect with the deep dorsal veins. The intermediate veins include the deep dorsal vein and the prostatic venous plexus, with the deep dorsal vein—located beneath the fascia penis—handling most of the penile venous drainage (28). ED can result from various issues, such as inadequate blood flow due to arterial insufficiency or venous leakage (29). A normal penile erection relies on proper vascular, neurological, and tissue responses (30). It is triggered by the integration of sensory stimuli and regulated by coordinated spinal activity (31). A schematic of the anatomical structure for normal flaccidity **(A)**, normal erection **(B)**, and erectile dysfunction **(C)** is shown in Figure 2. The regulation of penile erection involves multiple neurotransmitters, though the specifics are not yet fully understood.

### 1.2 International index of erectile function (IIEF)-15

Measurement and evaluation methods based on self-reports are practical tools for assessing sexual function in men and are periodically updated. Various questionnaires, alongside sexual function-related laboratory and clinical procedures have been introduced (32). The International Index of Erectile Function (IIEF) is one such tool. It is a multidimensional, abbreviated, and self-administered scale designed to assess several aspects of male sexual performance (33). Table 1 presents a simplified version adapted from the study by Rosick et al. (34). The standard version of the questionnaire consists of 15 items, evaluating five main areas of sexual function over a period of four or more weeks. These areas include erectile function (EF) with six items, sexual desire (SD) with two items, orgasmic function (OF) with two items, intercourse satisfaction (IS) with three items, and overall satisfaction (OS) with two items (35). The psychometric validity and reliability of the IIEF-15 have been established in earlier studies, making it a standard tool for assessing men's sexual function in both clinical and research settings without geographical limitations (36). The IIEF scale is commonly used in clinical studies of ED, facilitating the interpretation of study outcomes and helping monitor recovery levels after endovascular treatments or drug therapies.

**Table 1:** Simplified international Index of erectile function (IIEF-5) questionnaire and scoring guidelines.

Question Scoring	1	2	3	4	5
How do you rate your self-confidence regarding erection?	Very low	Low	Moderate	High	Very high
When you had an erection with sexual stimulation, how hard was your erection to penetrate?	Never/Almost never	A few times	Sometimes	Most-times	Always/Almost always
During intercourse, how many times were you able to maintain an erection after entering your partner?	Never/Almost never	A few times	Sometimes	Most-times	Always/Almost always
How difficult was it during sex to maintain an erection until the end of intercourse?	Extremely difficult	Very difficult	Difficult	Slightly difficult	Not difficult
When you had sexual intercourse, how many times was it satisfactory for you?	Never/Almost never	A few times	Sometimes	Most-times	Always/Almost always

Sum the responses to all five questions to get the total score.

**22–25**: No erectile dysfunction.

**17–21**: Mild erectile dysfunction.

**12–16**: Mild to moderate erectile dysfunction.

**8–11**: Moderate erectile dysfunction.

**5–7**: Severe erectile dysfunction.

## 2 Endovascular tools: a new therapeutic approach

The significance of penile blood supply for erectile function has been recognized for centuries. However, it was not until the early twentieth century that reports on penile vein closure emerged, and practical attempts to restore penile blood flow were first documented in 1973 (37). Endovascular treatments have since become promising options for addressing ED. These minimally invasive procedures improve blood flow to the penis and help restore erectile function (38). Although vascular microsurgeries were previously introduced, they were largely abandoned due to clinical complications. Recently, attention has shifted to novel technologies, such as the peripheral stent system, which are now at the forefront of treatment innovations (39).

As previously mentioned, a normal penile erection depends on proper vascular, neurological, and tissue responses (30). It is initiated by the integration of various sensory stimuli and regulated by coordinated spinal activity. Although the specific details of how neurotransmitters regulate penile erection are not fully understood, several key factors are essential for achieving an erection (24). These include a healthy nervous system, adequate arterial blood flow, well-functioning corpora cavernosa, and the ability to prevent venous blood outflow (40). The transition from a flaccid state to an erection involves a delicate balance between the central and peripheral nervous systems and the health of the penile vasculature (24).

The central nervous system responds to sexual stimulation by releasing dopamine from the hypothalamus, which triggers impulses through the spinal cord to initiate the erection process (41). Phosphodiesterase-5 (PDE5) compounds help return the penis to a flaccid state by breaking down cyclic guanosine monophosphate (cGMP) (42). Additionally, norepinephrine released from sympathetic neurons helps maintain flaccidity by activating alpha-1 G-protein receptors on smooth muscle cells in the cavernous sinusoids, leading to decreased intracellular calcium and relaxation of the smooth muscle cells (43).

### 2.1 Arterial interventions

The impact of adequate arterial flow on the initiation and stability of erections was recognized in the last century. In 1923, French surgeon René Leriche first described the relationship between aortiliac occlusion and ED (44). Later, in 1969, it was reported that 70% of men with aortiliac occlusion experienced ED, and relief from ED was noted after bilateral endarterectomy of the occluded internal iliac arteries (45). External iliac artery steal syndrome, a factor contributing to secondary ED, impedes the necessary arterial flow for erectile function (46). Pelvic steal syndrome, which affects approximately 27% of patients with ED, results in patients being potent at rest but impotent during sexual activity due to reduced blood flow to the penis, often referred to as “stealing” blood from the penile region (47–49).

**Figure 3:**
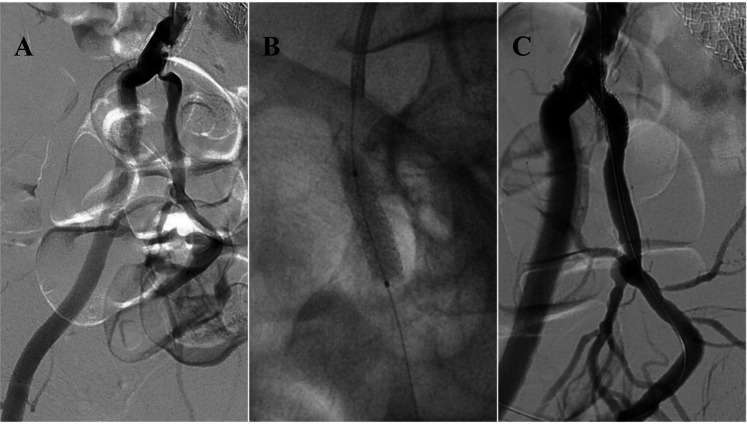
59-year-old male with peripheral arterial disease (PAD) and erectile dysfunction. Angiography reveals significant stenosis at the ostial part of the right internal iliac artery **(A)** Balloon-mounted stenting of the stenotic region, resulting in complete patency **(B,C)**. This figure is based on a case from the authors’ own clinical practice.

In a study by Rayt et al., it was found that adequate arterial flow is crucial for maintaining an erection. They observed new cases of ED in 17% of patients with unilateral internal iliac artery embolization and 24% of those with bilateral embolization before endovascular aortic aneurysm repair, although these results were not statistically significant (*P* = 0.33) (50). In the treatment of ED through arterial endovascular methods, percutaneous transluminal angioplasty (PTA) is a common approach, using a balloon catheter to widen narrowed arteries and improve blood flow to the penis (51). Another option is stent placement, which helps maintain artery patency (52) (Figure 3). Emerging techniques such as drug-coated balloons and gene therapy offer promising prospects for enhancing long-term vascular health and erectile function (53, 54). Recent studies have shown that drug-eluting stents (DES) achieve better outcomes than balloon dilation in patients with atherosclerotic ED, demonstrating improved long-term efficacy and lower restenosis rates (55).

With advancements in biotechnology, new techniques such as stenting have become common in endovascular treatments. Notably, the use of drug-eluting stents for ED was first investigated in 2009 with the Zotarolimus-Eluting stent trial. This study focused on patients with ED caused by PDE5 inhibitor-resistant internal pudendal artery stenosis (56). The findings were promising, showing significant improvement in erectile function for many patients, with no reported side effects (57).

Numerous ongoing studies are exploring various approaches to clinical interventions and treatment success, as summarized in Table 2. Rogers and Rocha-Singh emphasized the need for long-term, large-scale, controlled trials to confirm and generalize the efficacy of stents as a treatment option for arteriogenic ED (58).

**Table 2:** Characteristics of studies with endovascular interventions designs.

Study	Publication year	Cases No	Defect type (Arterial stenosis)	Applied approach	Outcomes
Schwartz et al. (74)	1988	40	The penis base arteries (58%) and IPA (31%)	Nonselective intracorporal papaverine angiography	Improvement without angiographic complications or adverse effects
Rosen et al. (75)	1990	195	Arteriogenic impotence (suspected).	Pudendal angiography	
Rogers et al. (58)	2012	30	IPA	PTA and DESs	At 6 months, 69.6% (95% CI: 47.1% to 86.8%) of per-protocol subjects showed IIEF improvement.
Wang et al. (76)	2014	20	Isolated penile artery stenoses (Common penile, Dorsal penile, and Cavernosal)	PTA	Clinical success (change in the IIEF-5 score ≥4 or normalization of erectile function was achieved in 15 (75%), 13 (65%), and 12 (60%) patients at one, three, and six months, respectively.
Wang et al. (77)	2016	22	Erectile dysfunction and isolated penile artery stenoses	PTA	At 8 months, 14 of 34 lesions (in 13 of 22 patients) had binary re-stenosis. At 1 year, sustained clinical success was achieved in 11 of 22 patients, and 8 of the remaining 9 patients who did not have binary restenosis achieved sustained clinical success.
Diehm et al. (39)	2018	50	Patients underwent endovascular revascularization for ED owing to >50% stenosis in 82 erection-related arteries	Standard balloon angioplasty (16%), drug-coated balloon angioplasty (27%), or DES (55%) implantation.	The overall IIEF-15 score improved in 32 (65%) of 49 patients. Change in the overall IIEF-15 score at 12 months was consistent among subgroups, except for elderly patients and those with hypertension who showed less improvement.

IPA, internal pudendal artery; PTA, percutaneous transluminal angioplasty; DES, drug-eluting stent.

**Figure 4:**
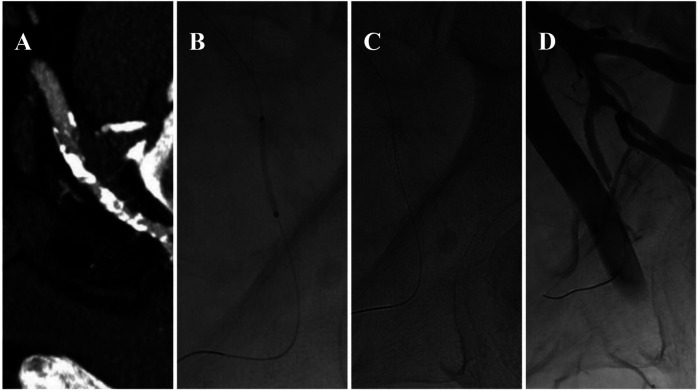
Endovascular treatment of left pudendal artery stenosis **(A)** staged angioplasty with a non-drug-coated balloon (1.5 mm × 20 mm, followed by 2 mm × 60 mm) **(B)**, placement of a 2.25 mm × 18 mm balloon-mounted stent **(C)**, post-procedure imaging demonstrating restored arterial patency **(D)** this figure is based on a case from the authors’ own clinical practice.

Additionally, balloon dilation of the internal pudendal artery has been studied as a treatment for ED secondary to peripheral arterial disease. Babaev and Jhaveri reported significant improvement in erectile function following internal pudendal balloon dilation. Furthermore, the placement of coronary stents of various sizes in the pudendal artery has yielded satisfactory results, with patients reporting improved erectile function during follow-up (59) (Figure 4).

In research by Schönhofen et al., the safety and clinical success of a novel Sirolimus-Eluting stent were evaluated in 100 male patients with arteriogenic ED or obstruction. These patients, who had atherosclerotic lesions in the arteries related to erection, were treated with angiolite BTK drug-eluting stents. The study assessed erectile function improvement using the International Index of Erectile Function (IIEF)-15 questionnaire at baseline and the third- and twelfth-months post-intervention. The results demonstrated significant improvement in erectile function without clinical side effects (60).

### 2.2 Endovascular embolization for venous endoleak in erectile dysfunction

Venous ED, resulting from impaired venous drainage of the corpora cavernosa, can be caused by various underlying factors. These include enlarged venous channels, degenerative changes or trauma to the tunica albuginea, structural modifications in the fibroelastic components, inadequate relaxation of trabecular smooth muscle, and acquired venous shunts (37).

Historically, the primary surgical interventions for venous leakage involved ligating both the superficial and deep dorsal veins, along with their collateral vessels (61). However, long-term success rates for these procedures generally did not exceed 25%, as reported in various studies. The limited success was attributed to difficulties in completely ligating all malfunctioning veins; small vein branches might be overlooked during surgery, and some proximal veins may be inaccessible due to exposure limitations (62). Additionally, the early development of collateral veins within the corpora cavernosa can contribute to the persistent failure of surgical ligation (63).

**Figure 5:**
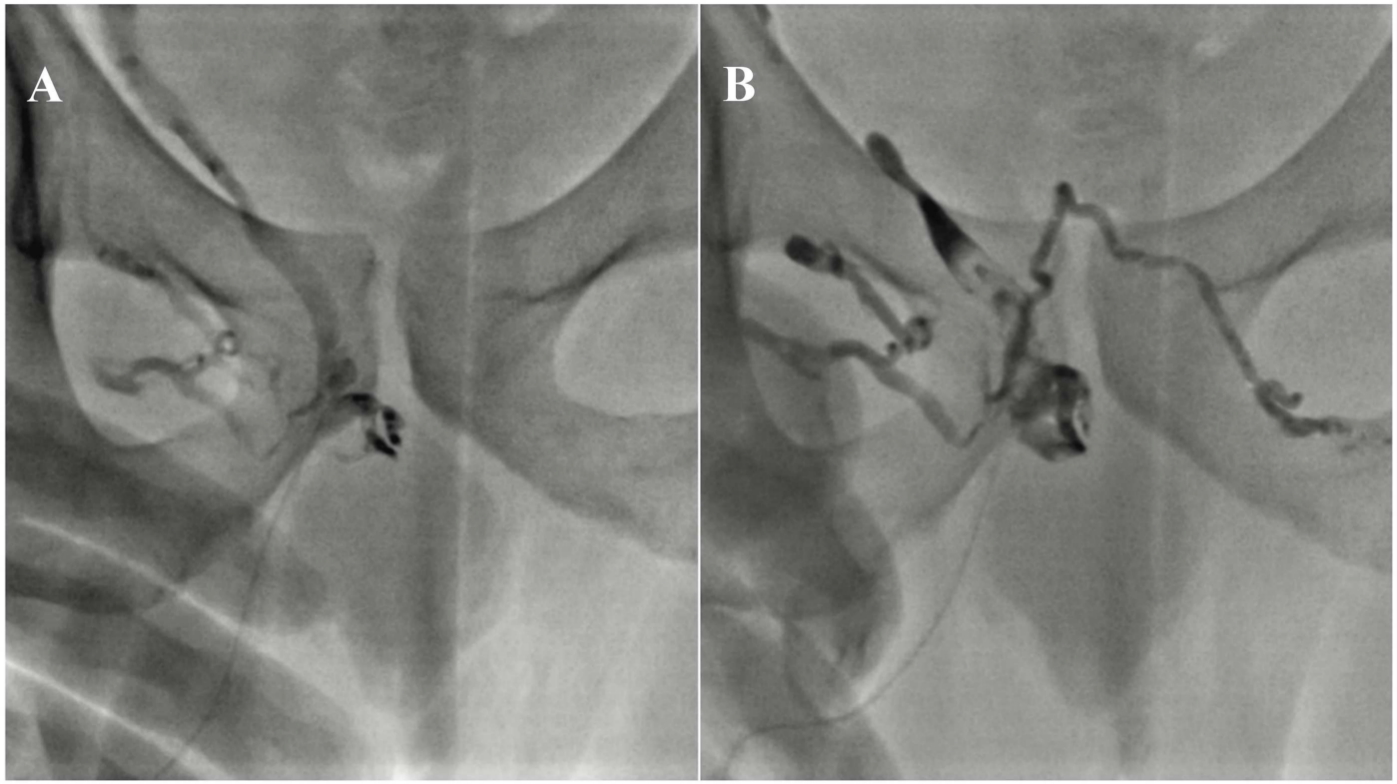
Pelvic venography following antegrade puncture of the deep dorsal vein demonstrates venous leakage through an incompetent right internal pudendal vein **(A)** post-embolization fluoroscopic image shows successful occlusion of one deep dorsal vein and both internal pudendal veins using a glue/lipiodol mixture **(B)** this figure is based on a case from the authors’ own clinical practice.

On the other hand, modern endovascular treatments, such as selective embolization, have been applied to patients with veno-occlusive dysfunction and are considered a safe and effective therapeutic option (64, 65) (Figure 5). In a 2015 study by Herwig and Sansalon, the effectiveness of pelvic vein embolization using the Aeroblock technique for treating ED caused by venous leakage was investigated. The study concluded that pelvic vein ablation is minimally invasive, cost-effective, and effective (66). Other techniques include ligating or rerouting veins to optimize blood flow dynamics in the penis (67). Recent evidence indicates that the success rates of venous endovascular treatments are comparable to those of arterial interventions, offering significant improvement in erectile function for properly selected patients. Diehm et al. reported that venous leak embolization via deep dorsal penile vein access demonstrated favorable safety and early efficacy (38, 68). In addition, certain patients with mixed arteriogenic and venogenic ED may require a combination of arterial and venous endovascular treatments to achieve optimal clinical outcomes. Recent evidence indicates that venous leak embolization performed after unsuccessful arterial revascularization can lead to significant functional improvement, highlighting the potential value of combined therapeutic strategies (69).

To determine who should be screened and which tool is appropriate, consider the following: Screening is necessary for individuals who have normal testosterone levels, no history of interventions in the urinary-genital area, and who do not respond to PDE5 inhibitors. For these individuals, the most common non-invasive screening tool for vascular ED is the intracavitary injection of a vasodilator combined with penile Doppler ultrasound imaging (70, 71). Doppler ultrasound is effective in detecting penile arterial insufficiency (PAI) and penile venous leakage (PVL), with a high sensitivity of 93.8% and good specificity of 77% (46, 72). Furthermore, in cases where penile Duplex sonography demonstrates vascular abnormalities, CT-angiography and/or CT-cavernosography should be performed for precise anatomical assessment and treatment planning (73).

## 3 Future perspectives and challenges

Endovascular therapeutic approaches for ED are rapidly evolving. Current research is focused on innovative therapeutic strategies and technologies designed to enhance procedural precision and long-term outcomes. Advances in imaging modalities, such as optical coherence tomography (OCT) and intravascular ultrasound (IVUS), are expected to enhance diagnostic accuracy and procedural performance.

The relationship between procedural success and complication rates remains a key consideration in endovascular therapy for ED. In general, higher procedural success is associated with fewer complications; however, even technically successful interventions cannot entirely eliminate risk. Adverse events, though relatively uncommon, underscore the importance of careful technique, standardized procedural protocols, and rigorous post-procedural evaluation. Continuous efforts to refine device design and optimize endovascular methods while minimizing complications are essential to enhance both the safety and effectiveness of these procedures.

Although procedural outcomes are promising, several challenges and limitations persist. Restenosis remains a major concern, particularly after balloon angioplasty or stent implantation in small-caliber arteries such as the internal pudendal artery. Wang et al. observed that approximately 41 percent of treated lesions exhibited binary restenosis within eight months, emphasizing the need for further optimization of device design and post-procedural management (74). Similarly, Rogers et al. and Diehm et al. reported that a subset of patients experienced limited improvement in erectile function despite technically successful interventions, suggesting that multifactorial mechanisms—such as neurogenic or psychogenic factors—may contribute to persistent dysfunction (39, 58). Another important limitation involves patient selection, as therapeutic efficacy appears highly dependent on vascular disease patterns and comorbidities. Endovascular therapy tends to be most effective in men with focal, angiographically confirmed stenoses of erection-related arteries—particularly the internal pudendal or penile arteries—who show inadequate response to phosphodiesterase type-5 inhibitors. Younger patients with limited systemic atherosclerosis and fewer comorbidities typically demonstrate greater functional improvement, whereas results are less favorable in those with diffuse, multilevel atherosclerotic disease or significant non-vascular etiologies.

The future of endovascular therapy for ED lies in the integration of technological innovation with personalized clinical strategies. Interdisciplinary and multidisciplinary collaborations—among urologists, vascular specialists, and interventional radiologists—are crucial for optimizing patient care and advancing the field. Long-term follow-up data are also necessary to evaluate the durability of treatment effects and to identify potential late complications. Emerging technologies will likely play a central role in addressing current limitations. The incorporation of artificial intelligence (AI) and machine-learning algorithms into imaging analysis and hemodynamic assessment could enhance lesion detection, automate quantification of vascular parameters, and support individualized treatment planning through predictive modeling. Furthermore, the development of next-generation stents, including drug-eluting, bioresorbable, and hemodynamically adaptive designs, may help reduce restenosis rates and improve long-term vessel patency. Integration of AI-based patient-selection algorithms combining clinical, imaging, and hemodynamic data may also help identify candidates most likely to benefit from these interventions, improving procedural efficiency and patient outcomes. Comprehensive pre-procedural evaluation, including penile Doppler ultrasound, CT-angiography, and/or CT-cavernosography, remains essential to complement these emerging technologies and ensure accurate patient selection and durable treatment success. Continued collaboration across disciplines and sustained innovation will be essential to define the optimal role of endovascular therapy within the broader management of ED.

## 4 Conclusion

Endovascular treatments offer a valuable therapeutic option for managing both arterial and venous types of ED, providing minimally invasive procedures with favorable efficacy and safety profiles. Although further research is needed to refine treatment protocols and expand the evidence base, these interventions show promise for improving the quality of life for men with ED. By leveraging innovative technologies and fostering collaborative efforts, endovascular approaches can continue to play a crucial role in the comprehensive management of ED.

Given the limited current reports on this global health concern, future studies should aim to enhance the understanding of ED-related mechanisms and evaluate treatment methods more rigorously. To achieve this, it is recommended that more case-control studies and clinical trials, involving both human and animal (*in vivo*) cases with larger sample sizes, be conducted to better generalize the results and assess the effectiveness of these interventions.
